# Correction: Impact of feralization on evolutionary trajectories in the genomes of feral cat island populations

**DOI:** 10.1371/journal.pone.0320028

**Published:** 2025-03-05

**Authors:** María Esther Nieto-Blázquez, Manuela Gómez-Suárez, Markus Pfenninger, Katrin Koch

In Fig 3, there is an error in panel A. The Manhattan plots should have not been identical. Please see the correct Fig 3 here.

**Fig 3:**
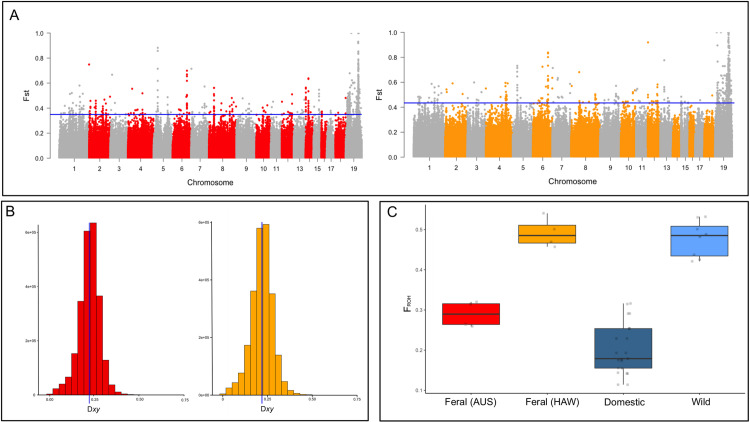
a) Manhattan plots of FST in 10-kb non-overlapping windows with the 98% FST threshold for Australian feral cats (in red) and Hawaiian feral cats (in orange); b) Distribution of absolute divergence values between Australian feral and domestic cats (in red) and Hawaiian feral and domestic cats (in in orange); c) Box plots of the inbreeding coefficients inferred from runs of homozygosity indicating the distribution of the per-individual number of ROH in different populations. Points indicate individuals.
