# Supplementary material for: Impact of feralization on evolutionary trajectories in the genomes of feral cat island populations
Source: PLoS One. 2024 Aug 13;19(8):e0308724. doi: 10.1371/journal.pone.0308724 (PMC11321585; doi:10.1371/journal.pone.0308724)
Supplement: S1 File — (DOCX) [file pone.0308724.s001.docx]

**Supporting Information**

**Impact of feralization on evolutionary trajectories in the genomes of feral cat island populations**

**María Esther Nieto-Blázquez^1^, Manuela Gómez-Suárez^1^, Markus Pfenninger^1^ and** [**Katrin Koch**](https://office.mailbox.org/appsuite/)**^2^**

*^1^Department of Molecular Ecology, Senckenberg Biodiversity and Climate Research Centre, Frankfurt am Main, Germany*

*^2^* *Department of Biodiversity, Conservation and Attractions, Former, Biodiversity and Conservation Science, Woodvale, Australia*

**Supplementary Methods**

**Table S1**. Individual’s sample information. Identification numbers, sample origin and cat group. Wild and feral cats sequenced data can be found in ENA project PRJEB40421. Domestic cats sequenced data can be found in ENA project PRJNA343389.

| **Sample ID** | **Origin** | **Cat group** |
| --- | --- | --- |
| D006A | Australia | Feral |
| D007A | Australia | Feral |
| D008A | Australia | Feral |
| D009A | Australia | Feral |
| HI007A | USA (Hawaii) | Feral |
| HI016A | USA (Hawaii) | Feral |
| HI017A | USA (Hawaii) | Feral |
| HI020A | USA (Hawaii) | Feral |
| FA699A | Germany | Wild |
| FB434A | Germany | Wild |
| FC779A | Germany | Wild |
| FC796A | Germany | Wild |
| FE383A | Germany | Wild |
| FF099A | Germany | Wild |
| FF567A | Germany | Wild |
| FF975A | Germany | Wild |
| SRR5359630 | USA (Iowa) | Domestic |
| SRR5359631 | Maui | Domestic |
| SRR5359632 | Jordan | Domestic |
| SRR5359633 | Italy | Domestic |
| SRR5359634 | Portugal | Domestic |
| SRR5359635 | Madagascar | Domestic |
| SRR5359636 | Denmark | Domestic |
| SRR5359637 | Oman | Domestic |
| SRR5359638 | South Korea | Domestic |
| SRR5359639 | Iraq | Domestic |
| SRR5359640 | Thailand | Domestic |

**Table S2.** Classification of evolutionary patterns in highly divergent windows with focus on the feral cat populations. The interpretation of the different patterns follows Feulner et al. [1]and Pfenninger et al.[2].

| **Derived feral cats** | **Ancestral domestic cats** | **Interpretation** |
| --- | --- | --- |
| Low T_D_ /low π | Inconspicious | Selective sweep/positive selection in feral cat populations |
| Inconspicious | Low T_D_ /low π | Relaxed selection in feral cat populations |
| Low T_D_ /low π | Low T_D_ /low π | Background selection in both |
| Inconspicious | Inconspicious | Divergence driven by random drift due to isolation |

**Supplementary Results**

**Table S3.** Highly differentiated genes found between Australian-domestic cats and Hawaiian-domestic cats.

| FERAL (AUS) | | | | | |
| --- | --- | --- | --- | --- | --- |
| Gene Names | **UniProt Entry Name** | **Protein names** | **Gene Length** | **Category** | **Annotation** |
| AHRR | M3WML1_FELCA | Aryl hydrocarbon receptor repressor | 581 | IMM | Xenobiotic metabolic process |
| LOC493964 | M3X1H6_FELCA | MAGE domain-containing protein | 311 | IMM | Related to immune system, melanoma-associated antigens |
| CLCN5 | M3W9W9_FELCA | Chloride channel protein | 816 | IMM | Immune system, neutrophil extracellular trap formation |
| HDAC8 | M3W3E4_FELCA | histone deacetylase (EC 3.5.1.98) | 351 | IMM | Neutrophil extracellular trap formation |
| TMEM164 | M3X1I3_FELCA | Transmembrane protein 164 | 351 | IMM | Positive regulation of ferroptosis |
| TMEM255A | M3WLM9_FELCA | Transmembrane protein 255A | 325 | IMM | [Response to bacterium](https://www.ebi.ac.uk/QuickGO/term/GO:0009617) |
| XIAP | M3WG83_FELCA | X-linked inhibitor of apoptosis | 454 | IMM | Inflammatory response |
| ICA1 | M3WI15_FELCA | Islet cell autoantigen 1 | 484 | INM | Related to immune response |
| COL4A2 | M3WLC3_FELCA | Collagen type IV alpha 2 chain | 1784 | MS | Response to activity |
| RAPGEF6 | M3VWQ8_FELCA | Rap guanine nucleotide exchange factor 6 | 1615 | MS | May play a role in fibroblast growth |
| SGCZ | M3XFV6_FELCA | Sarcoglycan zeta | 312 | MS | Cardiac muscle tissue development |
| HDAC4 | A0A2I2UVK3_FELCA | Histone deacetylase (EC 3.5.1.98) | 1083 | MS | Skeletal system and osteoblast development |
| OSTN | M3X933_FELCA | Osteocrin | 132 | MS | Bone-active molecule, has been shown in animals to be highly expressed in cells of the osteoblast lineage |
| RANBP10 | M3W0F8_FELCA | RAN binding protein 10 | 620 | MS | Broadly expressed, with highest levels in skeletal muscle |
| WWOX | M3XAJ7_FELCA | WW domain-containing oxidoreductase | 234 | MS | Osteoblast differentiation and skeletal system morphogenesis |
| HSD11B1 | M3X6E3_FELCA | Hydroxysteroid 11-beta dehydrogenase 1 | 292 | MS | Steroid hormone process (steroids play a role in tameness) |
| PLS3 | M3WA30_FELCA | Plastin 3 | 639 | MS | Bone development |
| MCTS1 | A0A2I2URS9_FELCA | Malignant T-cell-amplified sequence | 181 | MS | Regulation of growth |
| HS6ST2 | M3W0N1_FELCA | Heparan-sulfate 6-O-sulfotransferase (EC 2.8.2.-) | 608 | MS | Important for cartilage formation (bones and muscles) |
| GPC3 | M3W6M5_FELCA | Glypican 3 | 526 | MS | Important for cartilage formation (bones and muscles), hind limb development |
| ST8SIA4 | M3WTJ4_FELCA | ST8 alpha-N-acetyl-neuraminide alpha-2,8-sialyltransferase 4 | 359 | NEU | Nervous system development |
| EFNA5 | A0A337SUS6_FELCA | Ephrin A5 | 201 | NEU | Nervous system development |
| LOC101085186 | A0A337SMC8_FELCA | G-protein coupled receptors family 1 profile domain-containing protein | 313 | NEU | Sensory system, olfactory transduction |
| LOC101093132 | M3X1U1_FELCA | Olfactory receptor | 327 | NEU | Nervous system development |
| KCND2 | M3W8J5_FELCA | Potassium voltage-gated channel subfamily D member 2 (Voltage-gated potassium channel subunit Kv4.2) | 630 | NEU | Related to nervous system, Serotonergic synapse |
| NKAIN2 | M3VVL2_FELCA | Sodium/potassium-transporting ATPase subunit beta-1-interacting protein (Na(+)/K(+)-transporting ATPase subunit beta-1-interacting protein) | 85 | NEU | Brain development |
| DNAAF4 | A0A2I2V2S1_FELCA | Dynein axonemal assembly factor 4 | 376 | NEU | Nervous system development |
| ESPN | M3XDX1_FELCA | Espin | 881 | NEU | Sensory perception of sound |
| ZFYVE9 | M3XFS0_FELCA | Zinc finger FYVE domain-containing protein | 1424 | NEU | Expressed in frontal cortex |
| CCDC141 | M3WWW8_FELCA | Coiled-coil domain containing 141 | 1531 | NEU | Brain development |
| NLGN1 | M3WA79_FELCA | Neuroligin 1 | 823 | NEU | Plays a role in synapse function and synaptic signal transmission |
| TENM4 | M3W200_FELCA | Teneurin transmembrane protein 4 | 2769 | NEU | Neuron development |
| DCC | M3WHY3_FELCA | DCC netrin 1 receptor | 1446 | NEU | Nervous system development |
| SCAI | M3W014_FELCA | Suppressor of cancer cell invasion | 491 | NEU | Expressed in forebrain |
| DLG4 | M3VZ73_FELCA | Discs large MAGUK scaffold protein 4 | 699 | NEU | Neuron related |
| NLE1 | M3VUD4_FELCA | Notchless protein homolog 1 | 485 | NEU | Neuronal function and development |
| UNC45B | M3VUD5_FELCA | Protein unc-45 homolog B | 929 | NEU | Eye development (visual perception) |
| AP2B1 | M3WD64_FELCA | AP complex subunit beta | 908 | NEU | Postsynaptic neurotransmitter receptor internalization |
| CRX | D3VZV7_FELCA | Truncated cone-rod homeobox protein | 185 | NEU | Transcrition factor found upstream of several photoreceptor-specific genes, including the opsin genes >visual perception) |
| KCNB2 | M3WH41_FELCA | Potassium voltage-gated channel subfamily B member 2 | 911 | NEU | Related to action potential (nervous system) |
| SH3KBP1 | M3W166_FELCA | SH3 domain containing kinase binding protein 1 | 661 | NEU | Neuron development |
| IL1RAPL1 | A0A337RVK0_FELCA | Interleukin-1 receptor accessory protein-like 1 (X-linked interleukin-1 receptor accessory protein-like 1) | 696 | NEU | Neuron development |
| IL1RAPL2 | A0A337SMA0_FELCA | Interleukin 1 receptor accessory protein like 2 | 686 | NEU | Central nervous system development |
| CUL4B | M3WWH7_FELCA | Cullin 4B | 874 | NEU | Neuron development |
| GRIA3 | M3W9C0_FELCA | Glutamate receptor | 824 | NEU | Important in synaptic connection |
| THOC2 | A0A2I2V550_FELCA | THO complex subunit 2 | 1570 | NEU | Neuron development |
| SLC25A14 | M3W9M4_FELCA | Solute carrier family 25 member 14 | 325 | NEU | Brain mitochondria |
| TPH1 | M3X4L0_FELCA | tryptophan 5-monooxygenase (EC 1.14.16.4) | 527 | NEU/MS | Serotonin biosynthetic process and bone remodeling |
| CA5B | M3WVV0_FELCA | Carbonic anhydrase (EC 4.2.1.1) | 317 | NUT | Energy metabolism |
| FNIP1 | A0A337S3G6_FELCA | Folliculin interacting protein 1 | 1141 | NUT | Cellular response to starvation |
| NR2C2 | M3W0I7_FELCA | Nuclear receptor subfamily 2 group C member 2 | 596 | NUT | Cellular response to starvation |
| ECHDC1 | M3W8C4_FELCA | Ethylmalonyl-CoA decarboxylase 1 | 278 | NUT | Fatty acid beta-oxidation |
| OTC | M3WE39_FELCA | Ornithine transcarbamylase, mitochondrial (Ornithine carbamoyltransferase, mitochondrial) | 354 | NUT | Nutrition |
| ATP1B4 | A0A337SG11_FELCA | Sodium/potassium-transporting ATPase subunit beta | 357 | NUT | Pancreatic and bile secretation and endocrine system |
| KLHL1 | M3W2X8_FELCA | Kelch like family member 1 | 750 | REP | Tissue enhanced (endometrium) |
| RANBP17 | M3X4M4_FELCA | RAN binding protein 17 | 1215 | REP | Highly expressed in testis |
| PLTP | M3VXH3_FELCA | Phospholipid transfer protein | 401 | REP | Flagellated sperm motility |
| TET2 | A0A2I2UV82_FELCA | Methylcytosine dioxygenase TET (EC 1.14.11.80) | 1167 | REP | Plays a key role in epigenetic chromatin reprogramming during embryonic development |
| CDYL | M3WTM6_FELCA | Chromodomain Y like | 540 | REP | Spermatid development |
| ANKS1A | M3W4W8_FELCA | Ankyrin repeat and sterile alpha motif domain containing 1A | 1134 | REP | Plays a central role during spermatogenesis |
| TAF4B | M3WFL7_FELCA | TATA-box binding protein associated factor 4b | 869 | REP | Spermatogenesis (RI?) |
| RARA | M3WWH2_FELCA | Retinoic acid receptor alpha | 459 | REP | Estrogen signaling pathway |
| TSNAXIP1 | A0A2I2V498_FELCA | Translin associated factor X interacting protein 1 | 704 | REP | Spermatogenesis (RI?) |
| CTPS2 | M3W618_FELCA | CTP synthase (EC 6.3.4.2) (UTP--ammonia ligase) | 586 | REP | Expressed in endometrium |
| TEX11 | A0A5F5XLI4_FELCA | Protein ZIP4 homolog | 918 | REP | Male gonad development |
| KLHL13 | A0A2I2UAR4_FELCA | Kelch like family member 13 | 649 | REP | Tissue enhanced (endometrium) |
| SLC16A11 | Q53TS8 | Solute carrier family 16 member 11 | 326 | REP | Spermatogeneis |
| C2CD6 | Q53TS8 | C2 calcium dependent domain containing 6 | 393 | REP | Spermatogeneis |
| TFEC | M3WXG8_FELCA | Transcription factor EC | 436 | UV | Cellular response to heat |
| DNAJB6 | M3WN05_FELCA | DnaJ heat shock protein family (Hsp40) member B6 | 329 | UV | Regulation of cellular response to heat |
| TAF11 | M3WTA9_FELCA | TATA-box binding protein associated factor 11 | 211 | UV | DNA repair |
| SPRED1 | M3WGT9_FELCA | Sprouty related EVH1 domain containing 1 | 443 | UV | Positive regulation of DNA damage response |
| RAD51B | M3W844_FELCA | RAD51 paralog B | 277 | UV | [DNA repair](https://www.ebi.ac.uk/QuickGO/term/GO:0006281) |
| FAF1 | M3WEX0_FELCA | Fas associated factor 1 | 631 | UV | Binding to a heat shock protein, a protein synthesized or activated in response to heat shock |
| NRDC | M3X115_FELCA | Nardilysin convertase | 1159 | UV | Negative regulation of cold-induced thermogenesis |
| ANKRD44 | M3WA35_FELCA | Ankyrin repeat domain 44 | 999 | UV | DNA repair and recombination proteins |
| FAM168A | M3VZB0_FELCA | Family with sequence similarity 168 member A | 129 | UV | DNA repair |
| TAF15 | M3WP16_FELCA | TATA-box binding protein associated factor 15 | 545 | UV | DNA repair |
| SLC30A5 | M3W954_FELCA | Zinc transporter | 765 | else | Solute carrier family |
| TTC1 | A0A2I2UKY5_FELCA | Tetratricopeptide repeat domain 1 | 293 | else | Chaperones and folding catalysts |
| SNX13 | M3X2A7_FELCA | Sorting nexin 13 | 1182 | else | Intracellular protein transport |
| IQCM | M3X5G7_FELCA | IQ motif containing M | 499 | else |  |
| PFDN6 | M3XFX0_FELCA | Prefoldin subunit 6 | 129 | else | Protein folding |
| WDR46 | M3W1E4_FELCA | WD repeat domain 46 | 612 | else | Maturation of SSU-rRNA from tricistronic rRNA transcript |
| RGL2 | M3X6R7_FELCA | Ral guanine nucleotide dissociation stimulator like 2 | 778 | else | small GTPase-mediated signal transduction |
| RNF146 | M3WM22_FELCA | E3 ubiquitin-protein ligase (EC 2.3.2.27) | 356 | else | Wnt signaling pathway |
| EFCAB11 | A0A5F5Y179_FELCA | EF-hand calcium binding domain 11 | 214 | else | Calcium binding proteins |
| PITRM1 | M3VZZ9_FELCA | Pitrilysin metalloproteinase 1 | 1002 | else | Proteolysis |
| CELF2 | M3W2R1_FELCA | CUGBP Elav-like family member 2 | 495 | else | mRNA processing |
| GTF3C3 | A0A2I2UUL9_FELCA | Ral transcription factor IIIC subunit 3 | 849 | else | Transcription by RNA polymerase III |
| RFFL | M3VUD2_FELCA | Ring finger and FYVE like domain containing E3 ubiquitin protein ligase | 355 | else | Apoptotic process |
| CDC6 | M3X9D7_FELCA | Cell division control protein | 559 | else | Cell division |
| CENPT | M3WHQ9_FELCA | Centromere protein T | 558 | else | Mitotic cell cycle |
| NUTF2 | M3X0F5_FELCA | Nuclear transport factor 2 (NTF-2) | 127 | else | Nucleocytoplasmic transport |
| EDC4 | M3WHR0_FELCA | Enhancer of mRNA-decapping protein 4 | 1402 | else | Deadenylation-independent decapping of nuclear-transcribed mRNA |
| ZFP90 | A0A337SN43_FELCA | ZFP90 zinc finger protein | 93 | else | High level transcription switch |
| TNRC18 | M3VXL4_FELCA | Trinucleotide repeat containing 18 | 2918 | else | Chromatin binding |
| GRINA | M3WZV2_FELCA | Glutamate ionotropic receptor NMDA type subunit associated protein 1 | 443 | else | Negative regulation of endoplasmic reticulum stress-induced intrinsic apoptotic signaling pathway |
| AP1S2 | A0A337SLF1_FELCA | AP complex subunit sigma | 160 | else | intracellular protein transport, Human immunodeficiency virus 1 infection |
| ARL13A | M3X3K0_FELCA | ADP ribosylation factor like GTPase 13A | 273 | else | Receptor localization to non-motile cilium |
| SLC25A53 | M3X853_FELCA | Solute carrier family 25 member 53 | 306 | else | NAD transmembrane transport |
| NRK | A0A2I2UUX7_FELCA | Nik related kinase | 163 | else | Protein kinase activity |
| MORC4 | M3W4Z2_FELCA | MORC family CW-type zinc finger 4 | 936 | else |  |
| AMMECR1 | A0A5F5XCU5_FELCA | AMMECR nuclear protein 1 | 152 | else |  |
| PARD3B | M3X240_FELCA | Par-3 family cell polarity regulator beta | 1101 | else | Microtubule cytoskeleton organization/cell adhesion |

| FERAL (HAW) | | | | | |
| --- | --- | --- | --- | --- | --- |
| Gene Names | **UniProt Entry Name** | **Protein names** | **Gene Length** | **Category** | **Annotation** |
| C2 | A0A2I2V4P2_FELCA | Complement C2 (EC 3.4.21.43) (C3/C5 convertase) | 800 | IMM | Innate immune response |
| LOC111556169 | A0A5F5XY12_FELCA | Ig-like domain-containing protein | 276 | IMM | Adaptive immune response |
| RAB27A | A0A2I2UDJ9_FELCA | small monomeric GTPase (EC 3.6.5.2) | 221 | IMM | Immune response and/or colouring |
| RFTN2 | M3X6L4_FELCA | Raftlin family member 2 | 499 | IMM | Response to exogenous dsRNA, viral infection response |
| RASA2 | A0A2I2UFX2_FELCA | RAS p21 protein activator 2 | 854 | IMM | Inflammatory responses |
| NXF1 | M3WLB4_FELCA | Nuclear RNA export factor 1 | 618 | IMM | Immune reaction, antigen response |
| CTNNA3 | M3X1I9_FELCA | Catenin alpha 3 | 895 | IMM | Immune response, leukocyte transendothelial migration |
| LOC101082378 | A0A5F5Y820_FELCA | MAGE domain-containing protein | 371 | IMM | Related to immune system, melanoma-associated antigens |
| LOC493964 | M3X1H6_FELCA | MAGE domain-containing protein | 311 | IMM | Related to immune system, melanoma-associated antigens |
| TMEM164 | A0A337RTL2_FELCA | Transmembrane protein 164 | 258 | IMM | Positive regulation of ferroptosis |
| XIAP | M3WG83_FELCA | X-linked inhibitor of apoptosis | 454 | IMM | [Regulation of innate immune response](https://www.ebi.ac.uk/QuickGO/term/GO:0045088) |
| ZNF609 | M3WEX2_FELCA | Zinc finger protein 609 | 1411 | MS | Regulation of myoblast proliferation |
| WWOX | M3WHL3_FELCA | WW domain-containing oxidoreductase | 414 | MS | Osteoblast differentiation |
| MBTPS2 | A0A2I2USK0_FELCA | Membrane-bound transcription factor site-2 protease (EC 3.4.24.85) (Endopeptidase S2P) | 519 | MS | Bone maturation |
| PHEX | M3WSX1_FELCA | Phosphate regulating endopeptidase homolog X-linked | 749 | MS | Bone mineralization |
| HS6ST2 | A0A2I2UUD9_FELCA | Heparan-sulfate 6-O-sulfotransferase (EC 2.8.2.-) | 648 | MS | Important for cartilage formation (bones and muscles) |
| TENM2 | A0A2I2U127_FELCA | Teneurin transmembrane protein 2 | 2535 | NEU | Neuron development |
| LOC101085186 | A0A337SMC8_FELCA | G-protein coupled receptors family 1 profile domain-containing protein | 313 | NEU | Olfactory transduction |
| GABRA4 | M3WNN6_FELCA | Gamma-aminobutyric acid type A receptor subunit alpha4 | 554 | NEU | Neuronal cell projection |
| DNAAF4 | A0A2I2V2S1_FELCA | Dynein axonemal assembly factor 4 | 376 | NEU | Nervous system development |
| NDUFAF1 | M3WH95_FELCA | Complex I intermediate-associated protein 30, mitochondrial (NADH dehydrogenase [ubiquinone] 1 alpha subcomplex assembly factor 1) | 328 | NEU | Related to Thermogenesis |
| CAMTA1 | A0A337S2X9_FELCA | Calmodulin binding transcription activator 1 | 89 | NEU | Neuromuscular process controlling balance |
| PLCL1 | A0A2I2U9B4_FELCA | Phosphoinositide phospholipase C (EC 3.1.4.11) | 1040 | NEU | Regulation of synaptic transmission, GABAergic |
| GSK3B | A0A2I2U7S7_FELCA | [tau protein] kinase (EC 2.7.11.26) | 420 | NEU | Neural development, axon guidance |
| TENM4 | M3W200_FELCA | Teneurin transmembrane protein 4 | 2769 | NEU | Neuron development |
| TMEM223 | M3WSA9_FELCA | Transmembrane protein 223 | 202 | NEU | Nervous system development |
| DCC | M3WHY3_FELCA | DCC netrin 1 receptor | 1446 | NEU | Nervous system development |
| CNKSR2 | M3WVB2_FELCA | Connector enhancer of kinase suppressor of Ras 2 | 987 | NEU | Postsynaptic density |
| PTCHD1 | M3WQ84_FELCA | Patched domain containing 1 | 888 | NEU | Chemical synaptic transmission |
| PDZD11 | A0A337SDI3_FELCA | PDZ domain containing 11 | 179 | NEU | Neurotransmitter secretion |
| ARR3 | A0A5F5XHY2_FELCA | Arrestin 3 | 389 | NEU | Signal transduction, visual perception |
| IL1RAPL2 | A0A337SMA0_FELCA | Interleukin 1 receptor accessory protein like 2 | 686 | NEU | Regulation of presynapse assembly |
| CHRDL1 | M3W9T1_FELCA | Chordin like 1 | 379 | NEU | Regulation of synaptic plasticity |
| TRPC5 | A0A2I2UUK1_FELCA | Transient receptor potential cation channel subfamily C member 5 | 944 | NEU | Neural development, axon guidance |
| GRIA3 | A0A2I2U7P0_FELCA | Glutamate receptor | 894 | NEU | Important in synaptic connection |
| TENM1 | A0A2I2UQR9_FELCA | Teneurin transmembrane protein 1 | 2681 | NEU | Neuron development |
| ENOX2 | A0A2I2U5Z0_FELCA | Ecto-NOX disulfide-thiol exchanger 2 | 649 | NEU | [Ultradian rhythm](https://www.ebi.ac.uk/QuickGO/term/GO:0007624) |
| ARHGAP36 | A0A2I2V5E2_FELCA | Rho GTPase activating protein 36 | 522 | NEU | Group enriched (adrenal gland, brain, pituitary gland) |
| LOC101092501 | M3XBY2_FELCA | Olfactory receptor | 330 | NEU | **** |
| FRMD7 | A0A337ST73_FELCA | FERM domain containing 7 | 654 | NEU | Regulation of neuron projection development |
| AFF2 | A0A337SL81_FELCA | AF4/FMR2 family member 2 | 1314 | NEU | [Brain development](https://www.ebi.ac.uk/QuickGO/term/GO:0007420) |
| PCDH9 | Q9HC56 | Protocadherin 9 | 1050 | NEU | Forebrain development |
| HTR2C | A0A2I2UEL5_FELCA | 5-hydroxytryptamine receptor 2C (Serotonin receptor 2C) | 458 | NEU/NUT | Regulation of nervous system process and regulation of appetite |
| MED13L | M3W7X4_FELCA | Mediator of RNA polymerase II transcription subunit 13 | 2177 | NUT | Thyroid hormone signaling pathway |
| ACAA2 | A0A337SA65_FELCA | Acetyl-CoA acyltransferase 2 | 339 | NUT | Fatty acid metabolsim, nutrition |
| ABCA1 | M3W217_FELCA | ATP binding cassette subfamily A member 1 | 2261 | NUT | Cholesterol metabolism |
| CA5B | A0A2I2UW72_FELCA | Carbonic anhydrase (EC 4.2.1.1) | 332 | NUT | Energy metabolism |
| SERPINA7 | A0A5F5XK53_FELCA | Serpin family A member 7 | 399 | NUT | Thyroid hormone transport |
| STK26 | A0A337S2A9_FELCA | Serine/threonine kinase 26 | 354 | NUT | [Cellular response to starvation](https://www.ebi.ac.uk/QuickGO/term/GO:0009267) |
| KLHL1 | M3W2X8_FELCA | Kelch like family member 1 | 750 | REP | Tissue enhanced (endometrium) |
| MEIKIN | M3X4R4_FELCA | Meiotic kinetochore factor | 460 | REP | Homologous chromosome segregation |
| ADAM19 | M3WCF7_FELCA | ADAM metallopeptidase domain 19 | 909 | REP | Placenta development |
| SPEF2 | A0A2I2V240_FELCA | Sperm flagellar 2 | 2168 | REP | Sperm associated proteins |
| SYCP2 | M3W3I6_FELCA | Synaptonemal complex protein 2 | 1373 | REP | Fertilization and male genitalia morphogenesis |
| PLEK2 | A0A5F5XF53_FELCA | Pleckstrin 2 | 403 | REP | Most abundant in the thymus, large bowel, small bowel, stomach, and prostate. |
| GALNT8 | M3W513_FELCA | Polypeptide N-acetylgalactosaminyltransferase (EC 2.4.1.-) (Protein-UDP acetylgalactosaminyltransferase) | 588 | REP | Group enriched (intestine, testis) |
| SMS | M3VYH5_FELCA | Spermine synthase | 476 | REP | Sperm metabolic process |
| SMC1A | M3WJP4_FELCA | Structural maintenance of chromosomes protein | 1233 | REP | Oocyte meiosis |
| TEX11 | A0A5F5XLI4_FELCA | Protein ZIP4 homolog | 918 | REP | Male gonad development |
| IGSF1 | A0A2I2V365_FELCA | Immunoglobulin superfamily member 1 | 1243 | REP | Highly expressed in pancreas, testis |
| CDK7 | A0A337SJ00_FELCA | Cyclin-dependent kinase 7 (EC 2.7.11.22) (Cell division protein kinase 7) | 309 | UV | DNA repair |
| RAD51B | A0A337RXM3_FELCA | RAD51 paralog B | 350 | UV | [DNA repair](https://www.ebi.ac.uk/QuickGO/term/GO:0006281) |
| ATR | M3WP01_FELCA | Serine/threonine-protein kinase ATR (EC 2.7.11.1) | 2643 | UV | DNA damage checkpoint signaling |
| ATRX | M3WSD9_FELCA | DNA helicase (EC 3.6.4.12) (ATP-dependent helicase ATRX) | 2448 | UV | DNA repair |
| RPS6KA6 | M3WAV0_FELCA | Ribosomal protein S6 kinase (EC 2.7.11.1) | 744 | UV | DNA damage response |
| CRIP1 | M3WNB8_FELCA | Cysteine rich protein 1 | 77 | UV/REP/IMM | Intrinsic apoptotic signaling pathway in response to DNA damage/prostate gland stromal morphogenesis/immune response |
| RIOK2 | M3X9L1_FELCA | non-specific serine/threonine protein kinase (EC 2.7.11.1) | 552 | else | Ribosome biogenesis |
| SEC61A1 | A0A5F5XHY6_FELCA | SEC61 translocon subunit alpha 1 | 476 | else | Protein transport |
| BRPF3 | A0A337SFD8_FELCA | Bromodomain and PHD finger containing 3 | 872 | else | Chromatin remodeling |
| DPH6 | A0A2I2UIY9_FELCA | Diphthine--ammonia ligase (EC 6.3.1.14) (ATP-binding domain-containing protein 4) (Diphthamide synthase) (Diphthamide synthetase) (Protein DPH6 homolog) | 165 | else | Protein histidyl modification to diphthamide |
| KLHDC1 | A0A2I2U273_FELCA | Kelch domain containing 1 | 406 | else | Ubiquitin-dependent protein catabolic process |
| PPM1A | A0A337SV75_FELCA | protein-serine/threonine phosphatase (EC 3.1.3.16) | 486 | else | Protein export from nucleus/negative regulation of transforming growth factor beta receptor signaling pathway |
| CRIP2 | M3VV86_FELCA | Cysteine rich protein 2 | 208 | else | Related to hematopoiesis |
| TEDC1 | A0A2I2UZ24_FELCA | Tubulin epsilon and delta complex 1 | 418 | else | Positive regulation of smoothened signaling pathway |
| PITRM1 | A0A5F5XR05_FELCA | Pitrilysin metalloproteinase 1 | 978 | else | Proteolysis |
| ST6GALNAC3 | A0A337SBF5_FELCA | ST6 N-acetylgalactosaminide alpha-2,6-sialyltransferase 3 | 354 | else | Lipid metabolic process |
| GIPC2 | A0A5F5XNL8_FELCA | GIPC PDZ domain containing family member 2 | 296 | else | Membrane trafficking |
| GTDC1 | A0A2I2UKN3_FELCA | Glycosyltransferase-like domain-containing protein 1 | 496 | else | Glycosyltransferase activity |
| FBXO36 | M3WU13_FELCA | F-box protein 36 | 188 | else | Ubiquitin system |
| XRN1 | A0A337SIV3_FELCA | 5'-3' exoribonuclease 1 (EC 3.1.13.-) | 1707 | else | Ribosome biogenesis |
| UBE2E1 | A0A2I2UYS7_FELCA | Ubiquitin conjugating enzyme E2 E1 | 402 | else | ISG15-protein conjugation |
| NKIRAS1 | A0A5F5XTE2_FELCA | NFKB inhibitor interacting Ras like 1 | 289 | else | Regulation of tumor necrosis factor-mediated signaling pathway/Ral protein signal transduction |
| ZNF367 | M3WMW0_FELCA | Zinc finger protein 367 | 348 | else | Regulation of transcription by RNA polymerase II |
| KIF24 | M3WEZ9_FELCA | Kinesin family member 24 | 1427 | else | Microtubule-based movement |
| UBAP1 | A0A337S1R8_FELCA | Ubiquitin associated protein 1 | 472 | else | Ubiquitin-dependent protein catabolic process |
| REPS2 | A0A337S7T5_FELCA | RALBP1 associated Eps domain containing 2 | 650 | else | Endosomal transport |
| RIBC1 | M3WJP5_FELCA | RIB43A domain with coiled-coils 1 | 378 | else |  |
| ARL13A | M3X3K0_FELCA | ADP ribosylation factor like GTPase 13A | 273 | else | Receptor localization to non-motile cilium |
| TCEAL8 | M3W134_FELCA | Transcription elongation factor A like 8 | 117 | else |  |
| SLC25A53 | M3X853_FELCA | Solute carrier family 25 member 53 | 306 | else | NAD transmembrane transport |
| NRK | A0A2I2UUX7_FELCA | Nik related kinase | 163 | else |  |
| MORC4 | A0A337RZQ6_FELCA | MORC family CW-type zinc finger 4 | 967 | else |  |
| AMMECR1 | A0A337S3X6_FELCA | AMMECR nuclear protein 1 | 332 | else | Signaling proteins |
| STAG2 | A0A2I2UUV0_FELCA | Cohesin subunit SA (SCC3 homolog) (Stromal antigen) | 1231 | else | Cell division, chromosome segregation |

**SUPPLEMENTARY FIGURES**

**Figure S1**. 10-fold cross-validation error plot from ADMIXTURE.


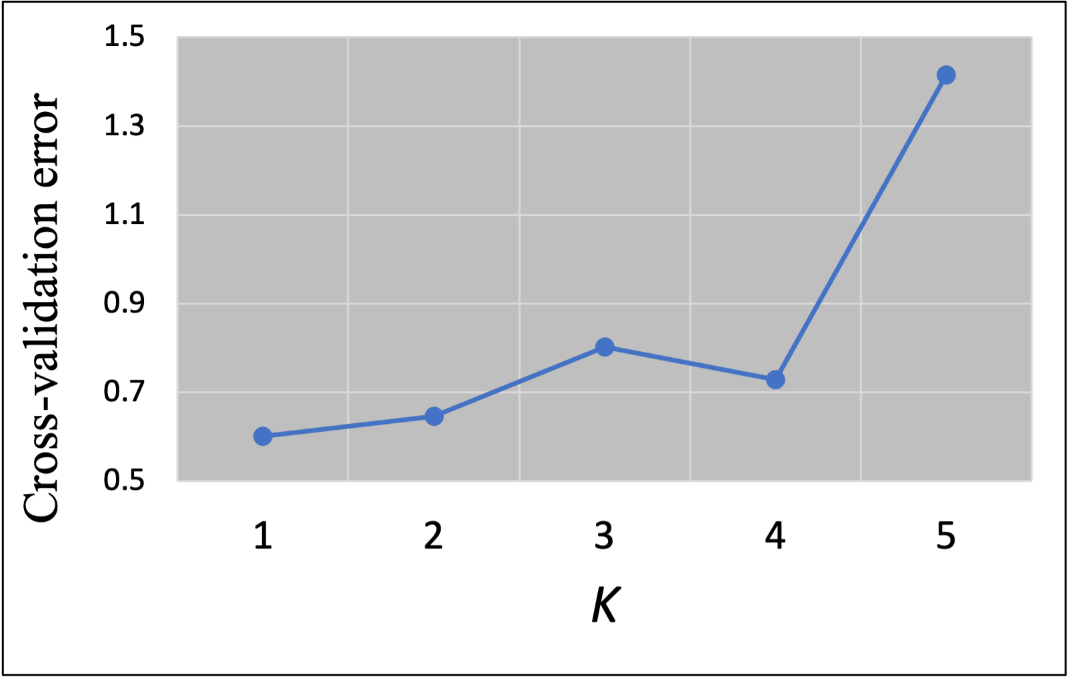


**Figure S2**. Stacked box plot of the inbreeding coefficients inferred from runs of homozygosity indicating the length of ROH per group.


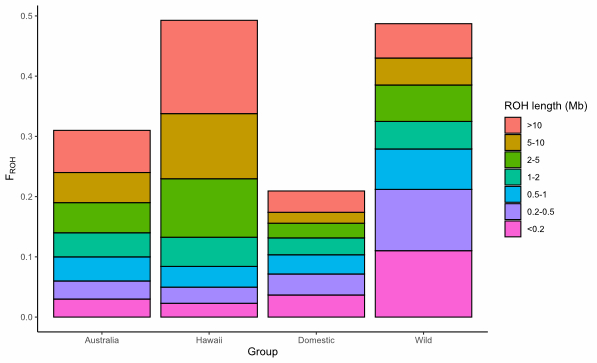


**Figure S3.** a-f) Bayesian paired t-test comparison of ROH by pairs of groups (feral Australia, feral Hawaii, domestic and wild cats.

1. Australia vs Domestic


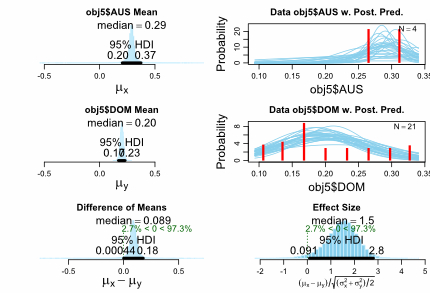


1. Australia vs Hawaii


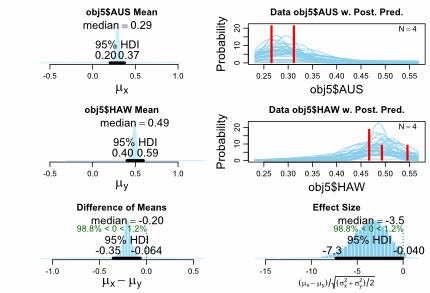


1. Australia vs Wild


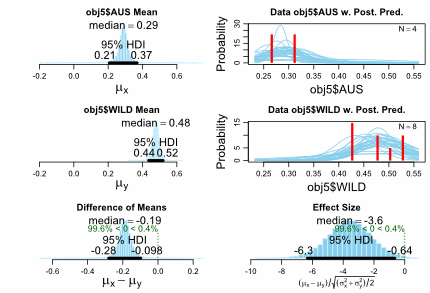


1. Domestic vs Wild


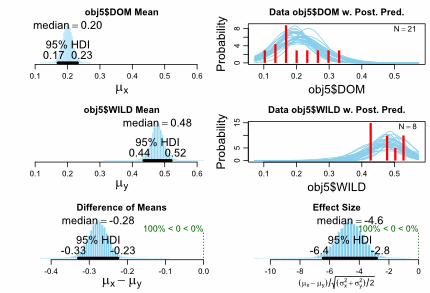


1. Hawaii vs Domestic


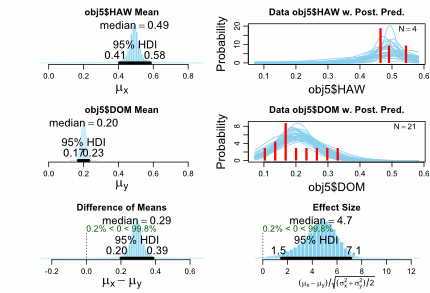


1. Hawaii vs Wild


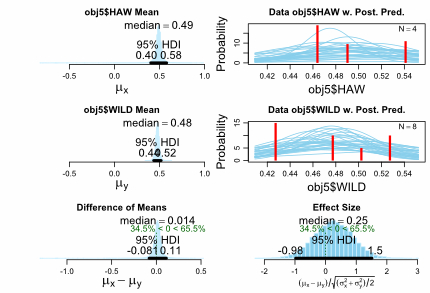


**Figure S4**. a-f) Bayesian paired t-test comparison of Watterson’s theta by pairs of groups (feral Australia, feral Hawaii, domestic and wild cats).

1. Australia vs Hawaii

**
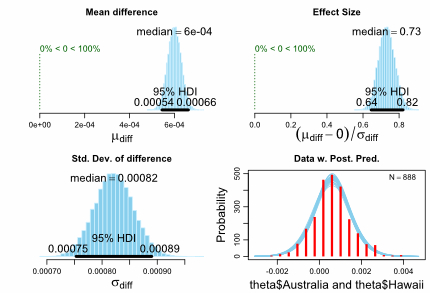
**

1. Australia vs Domestic


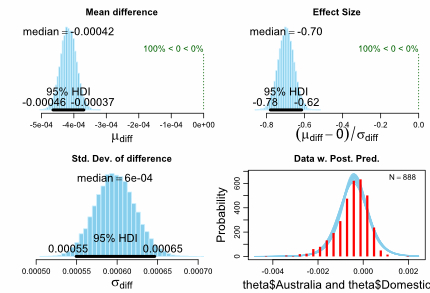


1. Australia vs Wildcat

**
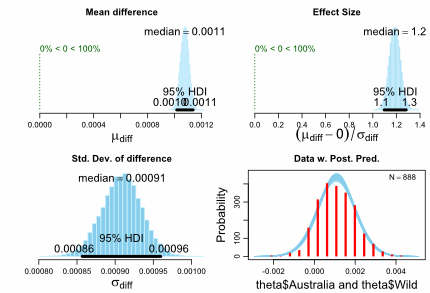
**

1. Hawaii vs Domestic


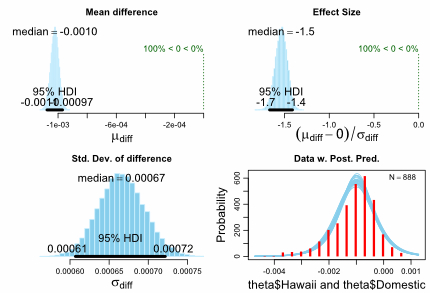


1. Hawaii vs Wildcat

**
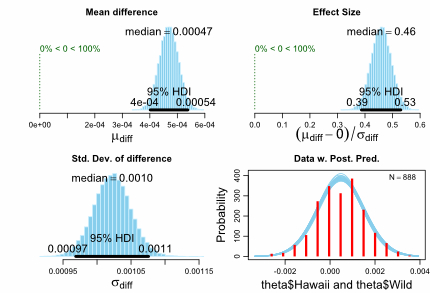
**

1. Domestic vs Wildcat


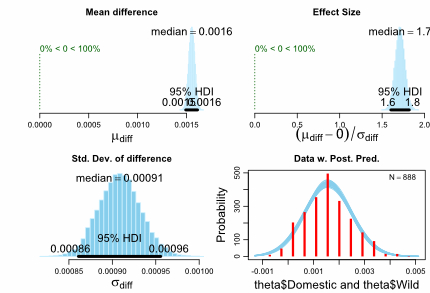


**Figure S5**. Recombination rates test and bootstrap results.

1. Australia feral cat


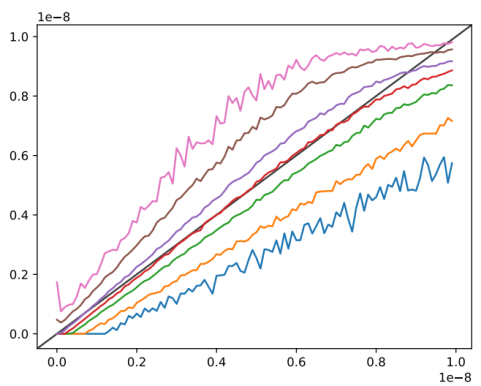

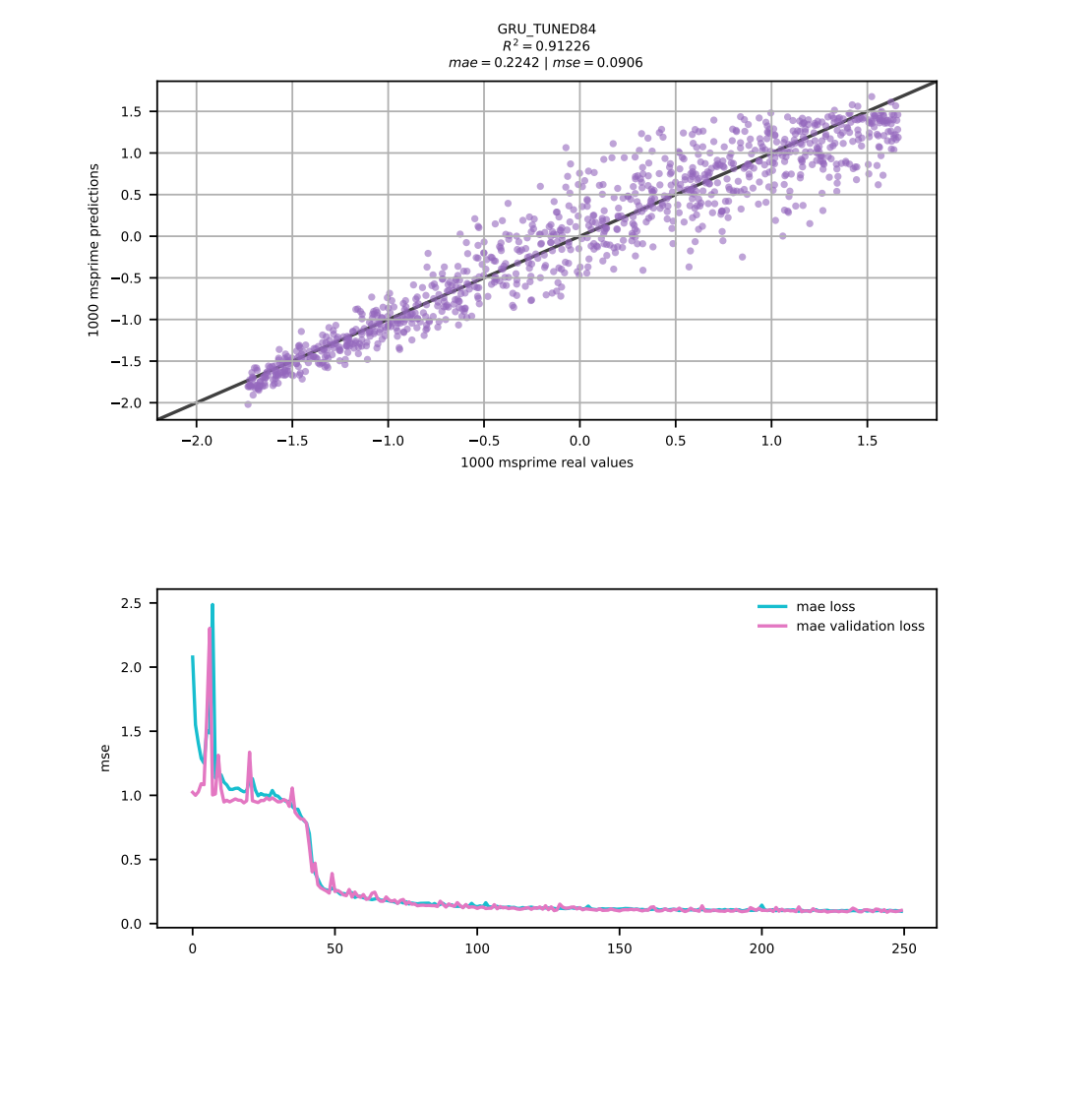


1. Hawaii feral cat


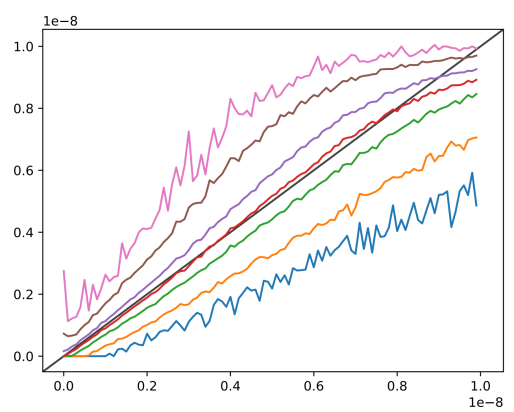

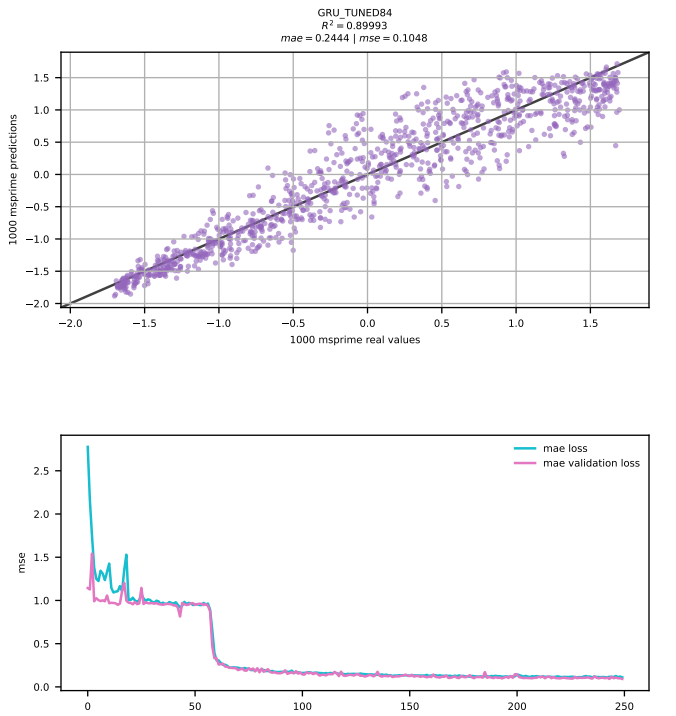


1. Domestic cat


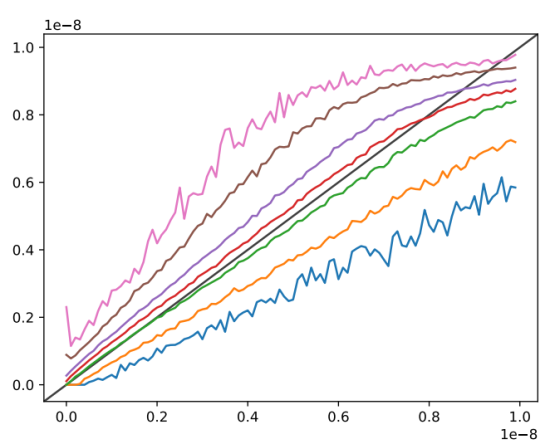

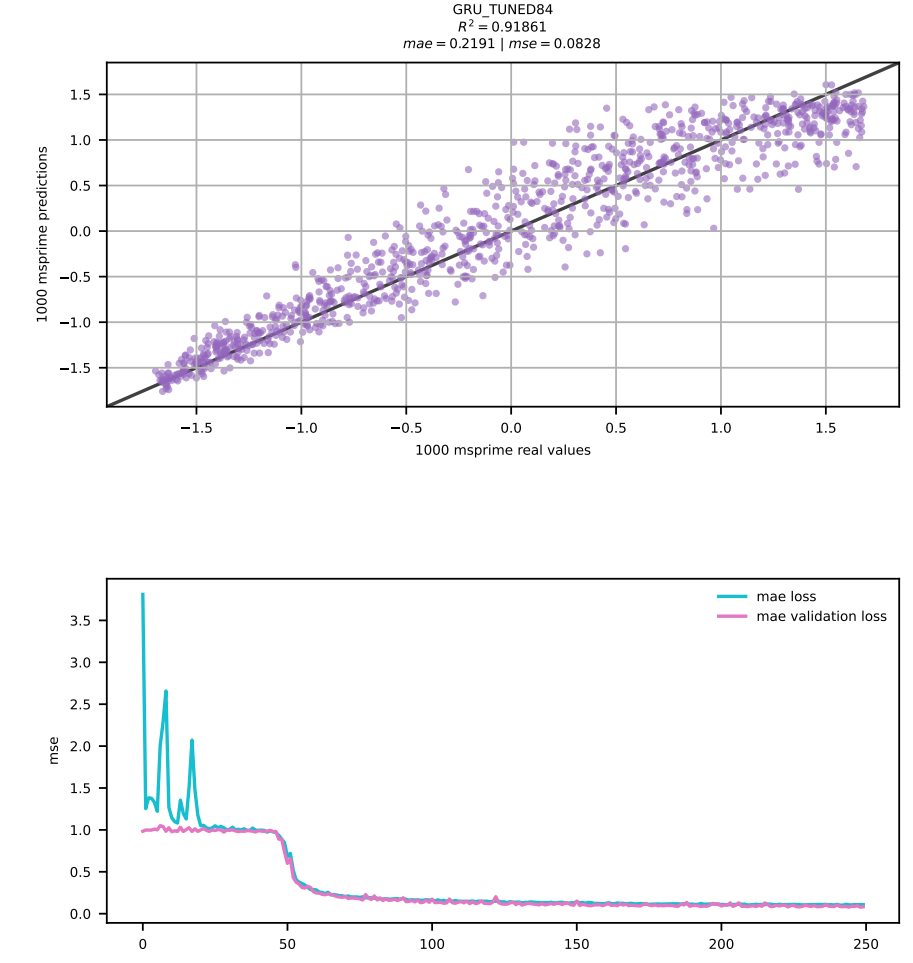


**Figure S6**. Average recombination rates across chromosomes for Australian and Hawaiian feral cats, and European domestic cats. Blue dots indicate the estimated recombination rate. Red bars represent the 95% CI (confidence intervals) for lower and higher bounds.


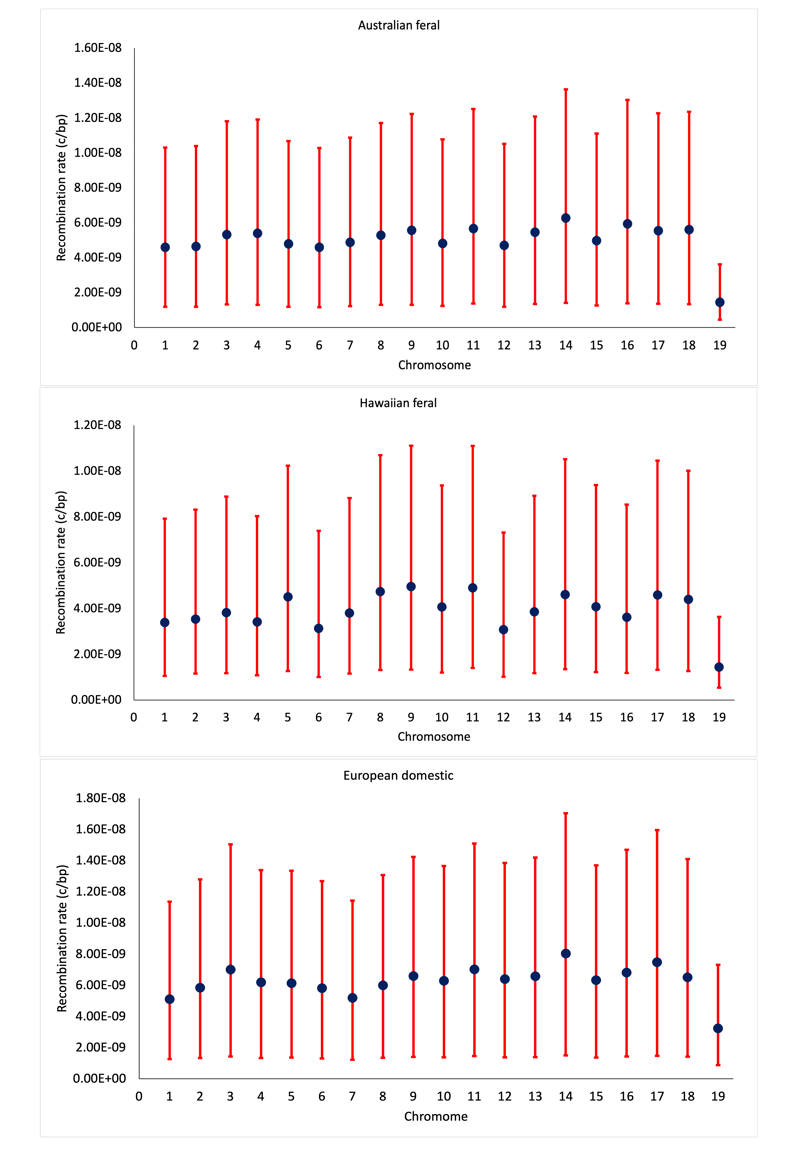


**Figure S7.** a-f) Bayesian paired t-test comparison of recombination rates by pairs of groups (feral Australia, feral Hawaii, domestic and wild cats).

1. Australia vs Domestic


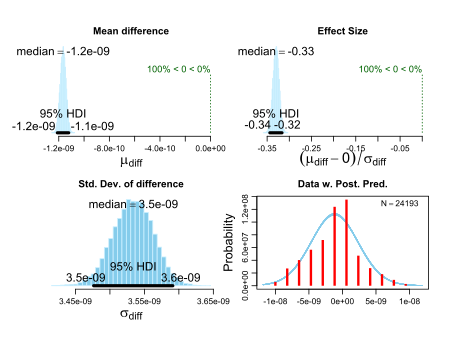


b) Australia vs Hawaii


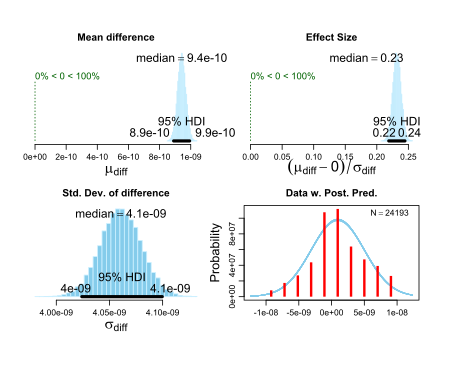


c) Australia vs Wild


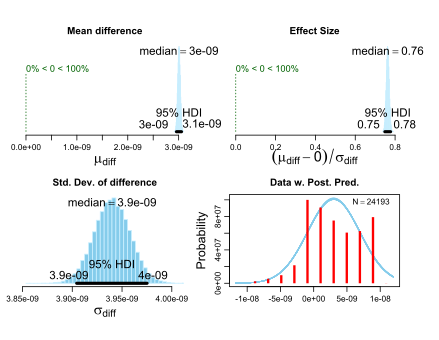


d) Domestic vs Wild


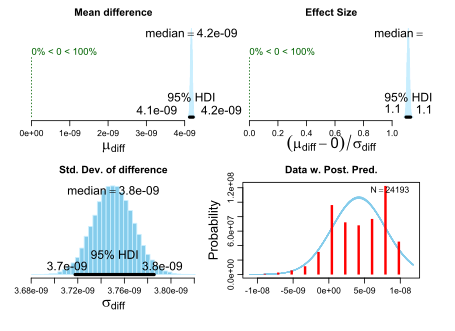


e) Hawaii vs Domestic


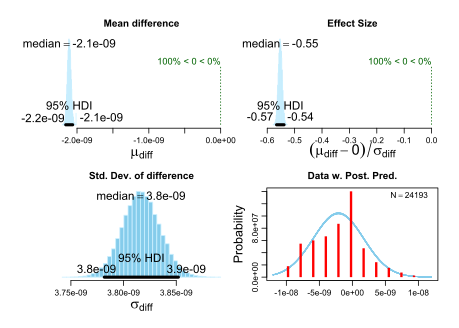


f) Hawaii vs Wild


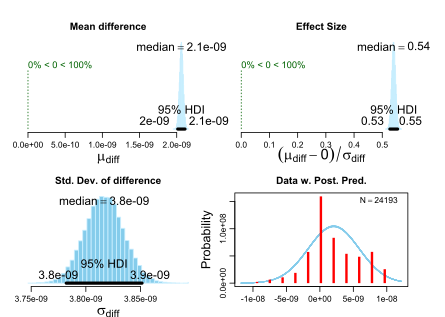


**REFERENCES**

1. Feulner PGD, Chain FJJ, Panchal M, Huang Y, Eizaguirre C, Kalbe M, et al. Genomics of Divergence along a Continuum of Parapatric Population Differentiation. PLOS Genet. 2015;11: e1004966. Available: https://doi.org/10.1371/journal.pgen.1004966

2. Pfenninger M, Patel S, Arias-Rodriguez L, Feldmeyer B, Riesch R, Plath M. Unique evolutionary trajectories in repeated adaptation to hydrogen sulphide-toxic habitats of a neotropical fish (*Poecilia mexicana*). Mol Ecol. 2015;24: 5446–5459. doi:https://doi.org/10.1111/mec.13397
