# Supplementary material for: Impact of feralization on evolutionary trajectories in the genomes of feral cat island populations
Source: PLoS One. 2024 Aug 13;19(8):e0308724. doi: 10.1371/journal.pone.0308724 (PMC11321585; doi:10.1371/journal.pone.0308724)
Supplement: Attachment — Submitted filename: Response to Reviewers v2.docx [file pone.0308724.s003.docx]

re: **Review 2** ID PONE-D-24-04531 entitled “Impact of feralization on evolutionary trajectories in the genomes of feral cat island populations”

**Journal Requirements:**

Please review your reference list to ensure that it is complete and correct. If you have cited papers that have been retracted, please include the rationale for doing so in the manuscript text, or remove these references and replace them with relevant current references. Any changes to the reference list should be mentioned in the rebuttal letter that accompanies your revised manuscript. If you need to cite a retracted article, indicate the article’s retracted status in the References list and also include a citation and full reference for the retraction notice.

Response: We have thoroughly reviewed the reference list and citations once more and have corrected some typos. We have found no evidence that any of the cited papers have been retracted.

**Response to reviewers**

**Additional Editor Comments:**

The Authors clearly took into account all comments satisfactorily, and only a few minor editing points remain. Once these have been resolved, the article should be accepted without problem.
I thank the Authors for taking the review process seriously and producing this much improved revision. I trust this will be a significant contribution to the field.

Response: We thank the Editor for acknowledging the authors effort to comply with the reviewers’ requirements. We are also grateful for their positive evaluation of our work.

We want to thank all 4 reviewers for their thorough and insightful comments on our manuscript.

**Review Comments to the Author**

**Reviewer #1**: (No Response)

**Reviewer #2**: The draft has been revised and improved. The main criticisms that have not been resolved have been adequately discussed. I have no further considerations but only the few listed below. Response: We appreciate the time and effort R2 has invested in providing valuable feedback, which has significantly improved the quality of our work.

I suggest checking the punctuation and verifying the correctness of the symbols (e.g. pi should be replaced with the correct symbol). The authors should verify to have introduced each symbol with the right definition before using it alone. Response: We have now checked the manuscript again for mistakes that we might have overlooked. We have now corrected mistakes in Line 183 (fixation index) and Line 383 (pi).

P17 L 404. I suggest the authors be careful in hypothesizing the same ship. Response: We agree with this comment. The sentence now reads as follows: “…*it appears likely that the two feral cat populations stem from similar ship introductions with global routes spanning* …” (Line 404).

P17 L414 The authors wrote: "… the North American connection
of the feral cat population remains so far historically unexplained." I suggest including some hypotheses due to the origin of sailors or the simultaneous presence in European ports of ships coming from different routes. Response: We acknowledge that providing well-formulated hypotheses for the observed phylogenetic pattern would be valuable. However, as this section is exploratory and not the primary focus of the paper, and due to the limited sample size, we prefer to avoid further speculation at this stage.

We agree that this is an interesting area for future research. We plan to address it more comprehensively in subsequent studies, where we can include a larger sample size and more historical information to conduct more in-depth phylogeographical analyses.

P19 L451 The authors wrote: “Our results point to low levels of inbreeding in domestic cats, which challenges the general assumption that domesticated species show considerable inbreeding". This affirmation is true but it has to be considered that domestic cats can also derive from admixture between different breeds, justifying a higher variability. Response: We thank the reviewer for this observation and have now incorporated this argument into the Discussion (Line 451-453).

**Reviewer #3**: From the revised manuscript, it is clear that the authors have taken my comments into account and made a concerted effort to correct the manuscript. I noticed only two small errors in the text:

Response: We appreciate the time and effort R3 has invested in providing valuable feedback, which has significantly improved the quality of our work.

- Line 437: Nucleotide diversity statistic (pi)... Please replace "pi" with the appropriate symbol for nucleotide diversity.

Response: We have now corrected this (Line 383).

- Line 454: "wild cats" is separated again; please correct this.
Response: We have now corrected this (Line 396 and 427).
